# Editorial: Molecular genetic testing and emerging targeted therapies for non-small cell lung cancer

**DOI:** 10.3389/fonc.2023.1308525

**Published:** 2023-11-23

**Authors:** Carlos Gil Ferreira, Marina Xavier Reis, Gilson Gabriel Viana Veloso

**Affiliations:** Thoracic Oncology Department, Instituto Oncoclínicas, Rio de Janeiro, Brazil

**Keywords:** NSCLC - lung adenocarcinoma - EGFR - ALK - BRAF - KRAS - RET - MET - PD-L1 - ROS1, NGS - next generation sequencing, molecular profiling, liquid biopsy, driver mutations, target therapy

In this Research Topic of Frontiers in Oncology, six interesting papers covering molecular targets, including *MET*, *ALK*, *EGFR*, *ERBB2*, and RET (Xu et al.; Xu et al.; Yang et al.; Sompallae et al.; Arora et al.; Zhang et al.), predicted response rates to target therapy, resistance mechanisms to first line treatments, in addition to how next generation sequencing (NGS) might predict tumor response. In fact, we’ve witnessed a revolution in the way we understand, diagnose, and treat non-small cell lung cancer (NSCLC) – particularly adenocarcinoma.

The development of molecular diagnosis and the resulting precision medicine have been based on three pillars. First, decades of advances in basic and translational research led to tumor biology clear up, with new signaling pathways and molecular targets being discovered, resulting in a wave of novel treatments for cancer patients based on crucial genetic alterations, the so-called actionable mutations. Secondly clinical practice underwent significant modifications with the adoption of NGS multigene panels in NSCLC, including both DNA and RNA-sequencing (Yang et al.), allowing fast and accurate molecular profiling. Thirdly those advances coupled to the chemistry advances led to the development of safe and effective tyrosine kinase inhibitors which were validated in well-designed pivotal registration trials. As a result, we have observed clear improvements in tumor response and survival upon the precision medicine framework in lung cancer.

Nevertheless, several challenges remain to be tackled if we are to expand the benefits of precision medicine in lung cancer to a global scale. Some barriers must be overcome in the coming years. Cost and sample quality issues limiting the widespread use of NGS; the lack of a clear guideline on how to couple liquid biopsy into the daily practice adding up to and complementing NGS tissue-based analysis and last, but not least the limited access to molecular testing and molecularly targeted drugs in low- and middle-income countries (LMIC).

Accuracy in the molecular diagnosis of lung cancer has evolved as novel technologies emerge. Pitfalls in previous molecular testing using immunohistochemistry (IHC), fluorescence *in situ* hybridization (FISH), and polymerase chain reaction (PCR) turned NGS as a resourceful tool for physicians (Yang et al.). Previously, DNA-sampling itself was considered enough to identify potential targets, however, it is becoming highly recommended to include RNA-sequencing on those tests (Zhang et al.). Not to mention that ideally molecular testing must be performed in both diagnostic sample and in a disease progression sample (1), aiming to identify potential mechanisms of resistance. Of note, the broad use of NGS can be cost-effective in some clinical scenarios (2, 3).

Concerning liquid biopsy, its use may enhance molecular profiling and assist in a more feasible treatment, as well as analysis of circulating tumor DNA, which, for instance, may guide duration of treatment (4), as well as anticipate the arise of resistant clones. Liquid biopsy can complement or substitute for tissue biopsy depending in the clinical scenario. Therefore, should we start to implement both liquid and tissue biopsies in all lung cancer cases and yet turn this into an affordable approach (5)?

Affordability is indeed an issue in LMIC as incorporation of novel test and drugs exponentially increase costs. The advent of precision medicine highlighted how inequity lung cancer care currently is, with disparities along several subsets of patients, especially among those who live in rural areas, Black patients, and patients derived from low and middle-income countries, in which molecular testing is almost nonexistent in poorer countries (6, 7). Inequity in access to precision medicine is multifactorial and should be tackled in a broader manner, including continuous medical education, awareness, and patient advocacy (8).

In continuum, with the advancements in precision medicine, the use of targeted therapy in early-stage NSCLC has been increasingly explored. The most recent example is the overall survival results from the ADAURA trial showing benefits with adjuvant osimertinib for EGFR-mutated NSCLC (9). Many promising studies with other targets such as ALK and RET in adjuvant settings are currently in development (9). The use of molecularly targeted drugs in the adjuvant setting raises the bar for the impact of precision medicine for lung cancer patients since those strategies can be curative.

In sum, the era of precision medicine in lung cancer has changed the landscape of the lung cancer treatment. There is no doubt that combined efforts to amplify access to these tests must include police-makers, collaborations between both diagnostic and pharmaceutical companies, and health care providers. Extending molecular testing to broader communities also depends on cost-effectiveness evaluations, high sensitivity rates, and real-world evidence on how affordable they can be. As technology advances, we must focus on overcoming the current existing barriers and increase access to molecular diagnosis, so that patients can yield better outcomes and quality of life (8).

## Author contributions

CF: Writing – original draft, Writing – review & editing. MR: Writing – original draft, Writing – review & editing. GV: Writing – original draft, Writing – review & editing.
